# Recurrent Steven-Johnson/Toxic Epidermal Necrolysis Overlap Syndrome

**DOI:** 10.7759/cureus.21364

**Published:** 2022-01-18

**Authors:** Juan F Toledo-Martinez, Ethel V Galdamez-Carcamo, Francisco J Somoza-Cano, Diego A Padilla-Mantilla, Karina L Alvarenga-Alvarado

**Affiliations:** 1 Internal Medicine, Cardio Center, San Pedro Sula, HND; 2 Internal Medicine, Universidad Catolica de Honduras, San Pedro Sula, HND; 3 Internal Medicine, Northeast Ohio Medical University, Cleveland, USA; 4 Internal Medicine, St. Vincent Charity Medical Center, Cleveland, USA; 5 Internal Medicine, Universidad de Los Andes, Bogota, DC, COL; 6 Dermatology, Hospital CEMESA, San Pedro Sula, HND

**Keywords:** honduras, carbamazepine, anticonvulsants, phenytoin, overlap disease, nikolsky's sign, sepsis, recurrent, toxic epidermal necrolysis (ten), steven-johnson

## Abstract

Steven-Johnson syndrome (SJS) and toxic epidermal necrolysis (TEN) are life-threatening mucocutaneous reactions that are predominantly drug-induced. Treatment varies depending on the severity, but even with accurate medical management, the mortality rate can be up to 50% in severe cases. Recurrent episodes with different agents are uncommon, but they have been reported in the literature. We present a case of a 30-year-old female presenting with recurrent SJS/TEN overlap syndrome complicated by sepsis after phenytoin use. Records revealed a previous episode after carbamazepine use one month prior to the current presentation and a first episode 23 years ago with an unknown medication. The offending agent was discontinued, the appropriate treatment was given, and the patient’s clinical status significantly improved. This case highlights the life-threatening manifestation of a mucocutaneous reaction. Prompt clinical assessment is paramount for patient survival.

## Introduction

Stevens-Johnson syndrome (SJS) and toxic epidermal necrolysis (TEN) are both potentially fatal mucocutaneous reactions characterized by an acute inflammatory process mediated by immune complexes and a hypersensitivity reaction. They are caused by triggering factors such as infections, connective tissue diseases, malignant neoplasms, radiotherapy, vaccines, and medications [1,2]. Both diseases belong to the same clinical entity but vary in severity. The diagnosis of SJS/TEN is clinical, and it is commonly associated with medications. Treatment consists of immediate withdrawal of the causative drug and supportive treatment. In addition, antibiotics and systemic steroids are also recommended in selected cases, as both diseases can be fatal if immediate measures are not taken [2].

## Case presentation

**Figure 1:**
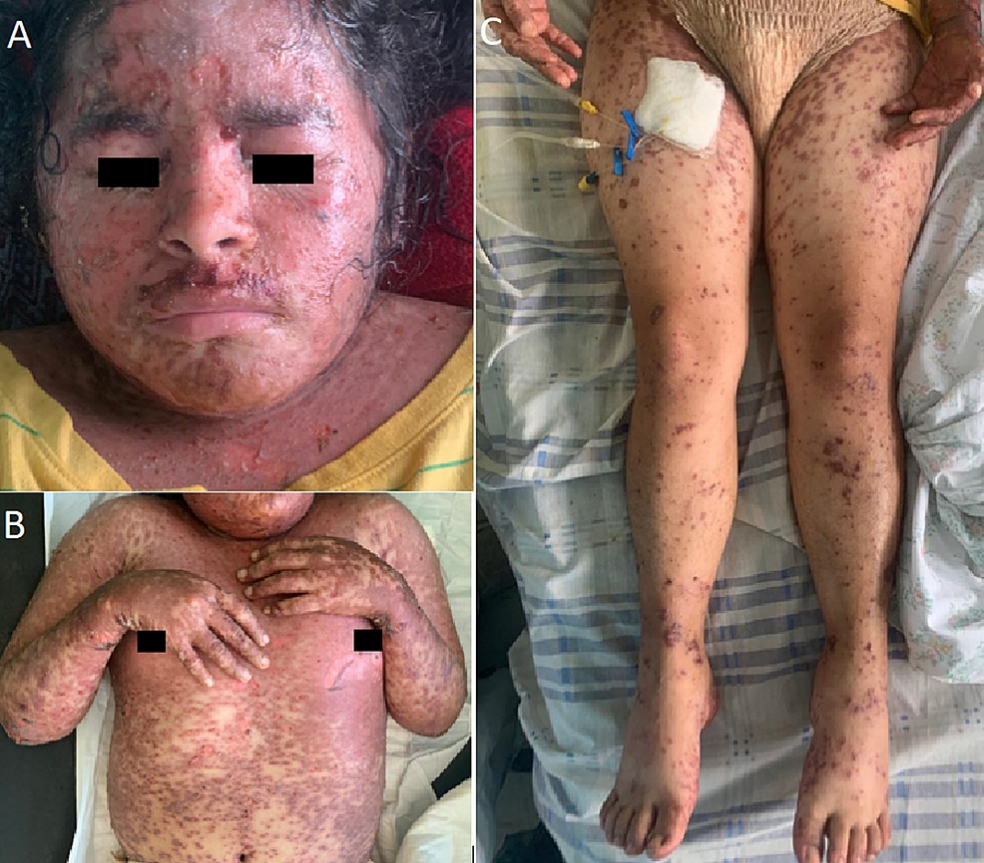
Anterior head, trunk, and legs on admission A maculopapular rash was seen throughout the body. Additionally, perioral lesions and scabs with a positive Nikolsky sign at neck was also documented (A). The anterior trunk presented with epidermal desquamation (B) and lower extremities showed mild pitting edema and vasculitic spots in addition to the rash (C).

**Figure 2:**
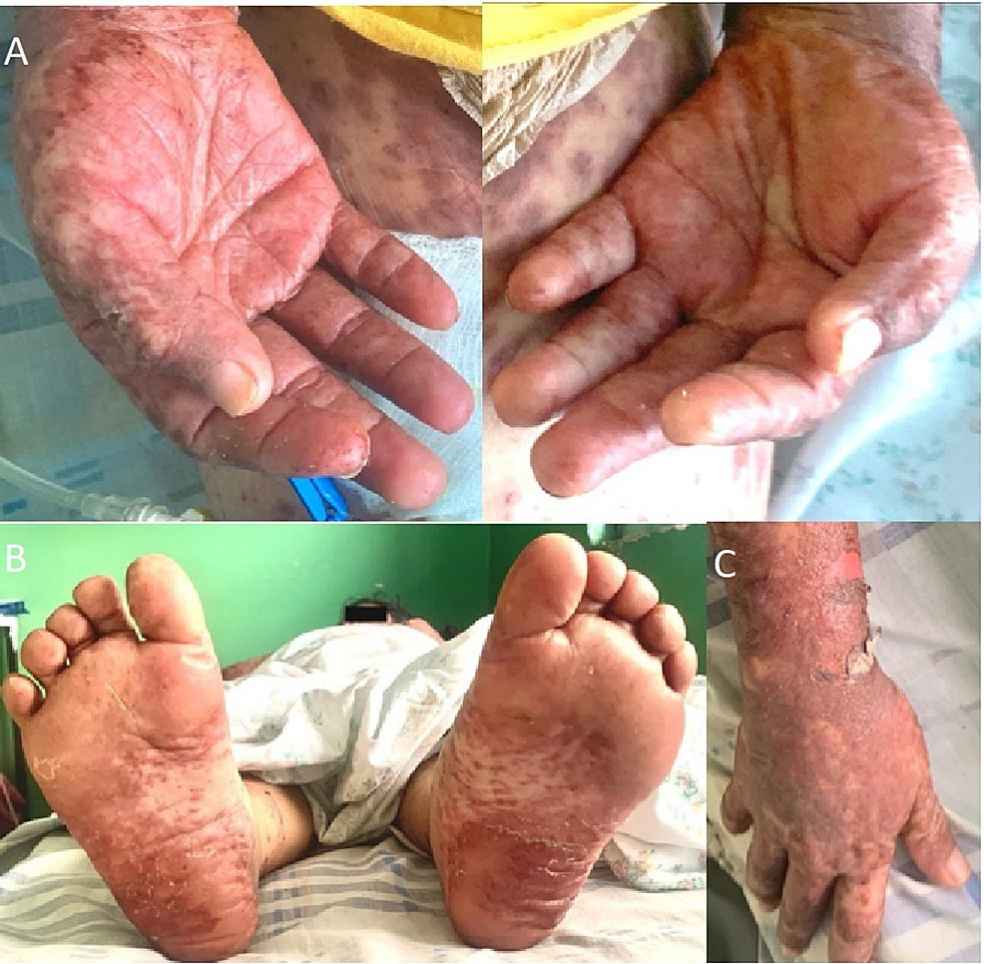
Hands, arms, and soles on admission Positive Nikolsky's sign in the right arm at the level of the wrist is seen, along with palmar desquamation (A,C). Furthermore, acral lesions with edema, purpuric spots and peeling of the dermis in the plantar region were observed (C).

**Figure 3:**
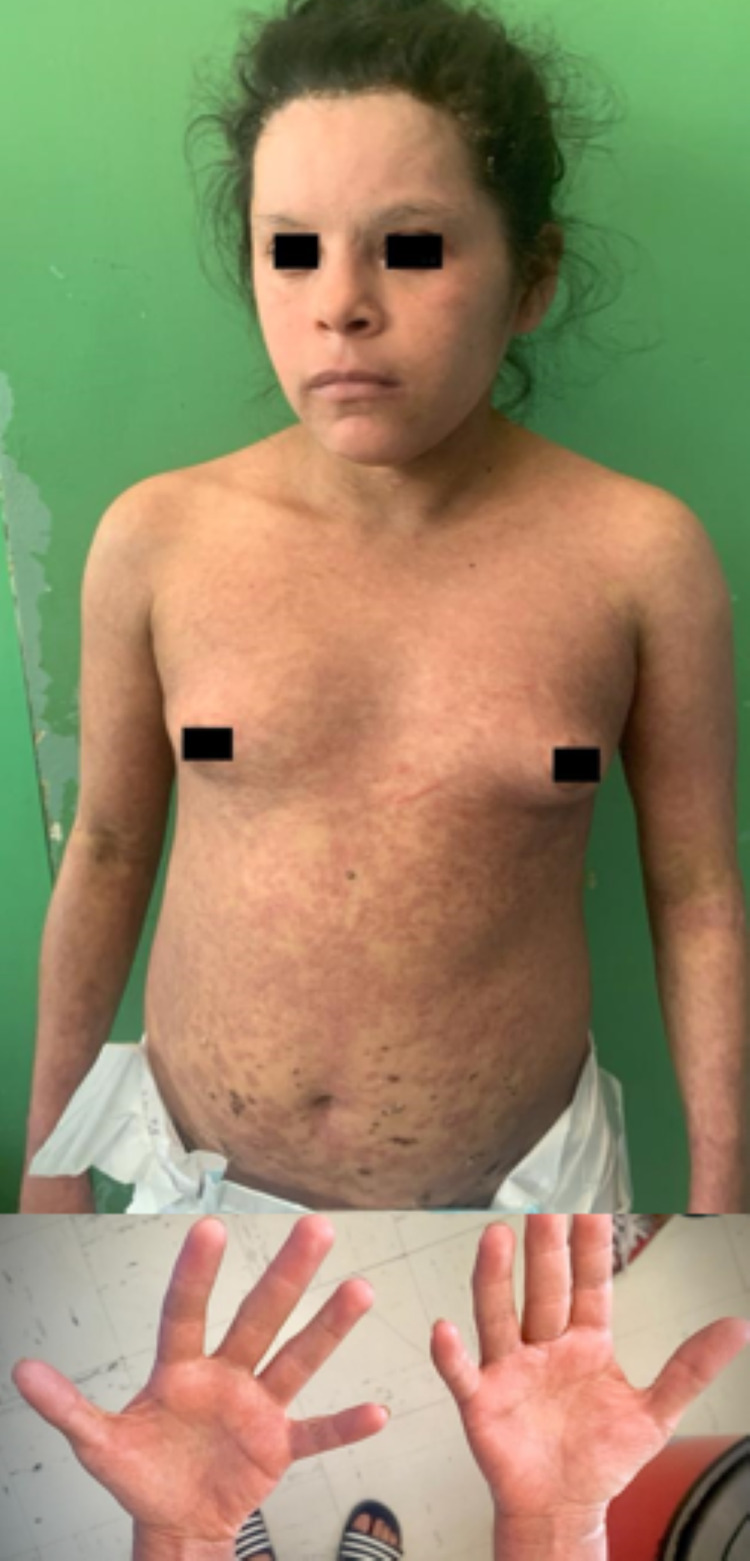
Lesions after 15 days of treatment The face and trunk were free of sloughing after 15 days of treatment, with re-epithelization of the dermis, hypopigmented areas, and minor desquamation in the thorax and extremities. The palms of the hands were unremarkable except for subtle areas of erythema without peeling.

A 30-year-old woman with a past medical history of epilepsy, type 2 diabetes mellitus, schizophrenia, and mental retardation with developmental delay presented with her caretaker to the emergency department of a Honduran public hospital complaining of a one-week history of skin lesions. Furthermore, her caretaker reported an intermittent fever of 39 ºC for 24 hours that improved after acetaminophen. Within 10 hours of fever onset, she developed asymmetrical skin lesions on her face and anterior trunk. Oral ulcers associated with dysphagia and difficulty with mild oral opening also developed at the same time. A review of systems was positive for dysuria. Her medications included phenytoin, valproic acid, metformin, biperiden, aripiprazole, and clonazepam. She had previously been admitted twice for SJS/TEN, the first time 23 years ago. Her caretaker did not recall specific trigger factors for the first occurrence, and medical records were unrevealing. Her second episode occurred 25 days prior to the date of the current presentation. It lasted for 10 days without serious complications. The identified trigger was carbamazepine, and her symptoms promptly improved after medication discontinuation. Phenytoin was started as a replacement. Her only known drug allergy was to erythromycin. On admission, the patient was vitally stable. A non-pruritic maculopapular rash on the face, neck, trunk, and extremities was documented. Furthermore, bilateral conjunctival injection with purulent secretion and positive Nikolsky's sign on the face, neck, and wrists with subsequent lip involvement (Figures 1A-1C, 2A-2C) and inflammation of the labia majora and minora were noted. These lesions covered at least 20% of her body using the "rule of palm" (the palm of the patient, without including fingers, is about 1% of the body). Initial workup was remarkable for 13,110 leukocytes/mm^3^, with 9,490 neutrophils/mm^3^, serum glucose of 216 mg/dl, BUN 10 mg/dl, creatinine 1.03 mg/dl, and sodium of 135 mmol/l. Additional electrolytes and liver function tests were within normal limits. Urinalysis showed 45-50 leukocytes per high power field (HPF), 35-50 erythrocytes per HPF, positive nitrites, and moderate bacteria. Additionally, C-reactive protein was elevated at 96 mg/L, as well as procalcitonin at 8.93 ng/ml and the erythrocyte sedimentation rate at 64 mm/h. A chest X-ray revealed no abnormalities. Supportive treatment, including analgesia and fluid resuscitation, was promptly started alongside methylprednisolone, diphenhydramine, ophthalmic prednisone, and ceftriaxone 2 g IV daily. Prophylactic anti-tetanus immunoglobulin was also administered. On day 3 of admission, she had worsening of the lesions on her neck and hands. The lesions had active purulent oozing and showed increasing sloughing. The lesions were treated with topical silver sulfadiazine and topical gentamicin. The mean arterial blood pressure dropped below 60 mm Hg. As a consequence, antibiotics were escalated to imipenem and vancomycin. In the following days, she showed clinical improvement with an appropriate response to treatment. Methylprednisolone was changed to oral prednisone, the urine and blood cultures were both negative after five days, and a repeat blood count showed 5,590 leukocytes/mm^3^, C-reactive protein improved to 14 mg/dl, and the rest of the complete metabolic panel was within normal limits. Fifteen days after the initial presentation, the skin lesions had re-epithelialized (Figure 3).

The patient complained of pruritus in the erythematous areas. Prednisone was progressively tapered as an outpatient and the patient's caretaker was advised on permanent phenytoin discontinuation. She made a full recovery thereafter.

## Discussion

SJS and TEN are two diseases on the same continuum manifesting in different degrees of severity. Mucous membranes are affected in over 90% of patients, and usually at two distinct locations. SJS affects less than 10% of the body surface, while TEN involves epidermal detachment of more than 30% of the body surface. The in-between range is called an SJS/TEN overlap [1,2]. Drugs remain the most common trigger for SJS and TEN. The reaction usually appears within the first two months of exposure to the offending agent, but the typical exposure period before the epidermal necrosis is four days. These two entities constitute a hyperacute life-threatening pathology, histologically identical to each other [3,4].

The etiology of both entities is multifactorial, with drugs being one of the most frequent etiological agents [5]. In SJS, a strong association with specific drugs is observed in up to 50% of cases, whereas in the TEN, this association is found in up to 80% of cases [6]. These syndromes are strongly linked to anticonvulsants, as seen in our case, but they can also be caused by other medications such as penicillin, sulfonamides, and nonsteroidal anti-inflammatory drugs [7]. The most common offending anticonvulsants are phenytoin, carbamazepine, oxcarbazepine, phenobarbital, and lamotrigine [8]. In our case, phenytoin was determined to be the causative agent as it had been started 15 days prior to the symptom onset, and her clinical status promptly resolved after discontinuation. Moreover, the Naranjo Adverse Drug Reaction Probability scale for adverse drug reactions was 6 points, making the diagnosis of SJS/TEN secondary to phenytoin probable [9].

The initial clinical presentation is usually nonspecific until the mucocutaneous lesions appear. SJS/TEN begins with a prodrome of fever and influenza-like symptoms one to three days before the development of mucocutaneous and skin lesions [10]. The cutaneous eruptions usually begin with ill-defined erythematous macules and papules that coalesce along with the presence of atypical target lesions. Vesicles and bullae form as the disease progresses, and within days the skin begins to slough [11]. The skin’s final appearance resembles that of an extensive burn injury [12]. Mucosal involvement occurs in approximately 90% of cases of SJS/TEN, which may include the internal organs’ mucosal lining and may precede or follow the skin eruption. Fever, malaise, myalgia, and arthralgia are present in most patients [10-12]. 

The treatment for SJS/TEN is usually supportive but varies depending on the severity and local guidelines [10]. The most frequent acute complications include sepsis, as seen in our patient, and dehydration due to fluid loss. Severe manifestations of these complications account for a great degree of morbimortality. Also, pulmonary and gastrointestinal complications due to mucosal necrosis may be present as well [1,2]. The most important step forward is to discontinue the offending agent and prompt documentation of the findings to prevent re-exposure. The prognosis depends on the severity of the disease and the associated medical conditions. Mortality is about 10% for SJS and up to 50% for TEN [1,2,5,6]. If the patient’s clinical scenario has worsening severity or its status fails to improve after the appropriate treatment is started, additional immunosuppressants and transfer to a burn unit should be considered [10,12].

The inflammatory process may last up to six weeks, and injury recovery usually leaves no scars [13,14]. The improvement in the skin lesions our patient had on admission was observed on day 15 after symptom onset. Even though biopsy is the gold standard for SJS/TEN, these entities can be clinically diagnosed and biopsies can be avoided if the clinical presentation is unclouded [10]. In our case, the symptoms and lesions were consistent with these types of toxicodermas, and the public hospital where she was admitted had the inability to deliver a biopsy result promptly. Our patient continued to have mild hypopigmented skin lesions with desquamation and re-epithelialization of the dermis, but the symptoms fully resolved after six weeks.

Finally, multiple recurrences of SJS/TEN are exceedingly rare. A 10-year population-based cohort study by Finkelstein et al. reported multiple recurrences happening in only 1.4% of cases (an incidence rate of 16 episodes per 1000 person-years and a median time to recurrence of 315 days) [15]. Our patient had a recurrent episode less than a month after a previously resolved SJS, and it was the third time overall presenting with this disease continuum.

## Conclusions

Recurrent SJS/TEN overlap is a rare entity, uncommonly seen in clinical practice. Immediate clinical recognition is of the utmost importance for drug discontinuation and the prevention of fatal complications such as sepsis. Additionally, a thorough clinical history and documentation are essential to reduce the re-exposure risk to the toxic insult. Multiple immunosuppressants and transfer to a burn unit should be considered in severe or refractory cases.
